# Extracellular Matrix in Regulation of Contractile System in Cardiomyocytes

**DOI:** 10.3390/ijms20205054

**Published:** 2019-10-11

**Authors:** Natalya Bildyug

**Affiliations:** Institute of Cytology, Russian Academy of Sciences, St-Petersburg 194064, Russia; nbildyug@gmail.com

**Keywords:** cardiomyocyte, actin contractile system, extracellular matrix

## Abstract

The contractile apparatus of cardiomyocytes is considered to be a stable system. However, it undergoes strong rearrangements during heart development as cells progress from their non-muscle precursors. Long-term culturing of mature cardiomyocytes is also accompanied by the reorganization of their contractile apparatus with the conversion of typical myofibrils into structures of non-muscle type. Processes of heart development as well as cell adaptation to culture conditions in cardiomyocytes both involve extracellular matrix changes, which appear to be crucial for the maturation of contractile apparatus. The aim of this review is to analyze the role of extracellular matrix in the regulation of contractile system dynamics in cardiomyocytes. Here, the remodeling of actin contractile structures and the expression of actin isoforms in cardiomyocytes during differentiation and adaptation to the culture system are described along with the extracellular matrix alterations. The data supporting the regulation of actin dynamics by extracellular matrix are highlighted and the possible mechanisms of such regulation are discussed.

## 1. Introduction

Cardiomyocytes (CMs) are heart muscle cells, which are responsible for contractility. Their highly organized contractile apparatus is considered to be a stable system. However, the processes of differentiation, as well as adaptation to culture conditions in these cells, involve significant rearrangements of their contractile structures.

In normal adult myocardium, the contractile apparatus is composed of actin-based myofibrils with the sarcomeric actin represented by α-skeletal and α-cardiac isoform the latter being predominantly expressed [1,2,3]. Early cardiogenesis, as well as stem cell differentiation towards CMs [4,5] are both accompanied by the remodeling of actin structures, wherein α-smooth muscle actin isoform is transiently expressed with its sequential replacement by α-skeletal and α-cardiac actin as development proceeds [2,6,7]. Similar processes are observed in cell adaptation to the culture system, where CMs undergo the reversible rearrangement of their contractile apparatus with the conversion of typical myofibrils into structures of non-muscle type and the loss of contractility. This phenomenon is accompanied by the transient replacement of the inherent α-cardiac actin with α-smooth-muscle isoform [2,8,9,10,11]. The following down-regulation of α-smooth muscle actin goes along with the expression of cardiac isoform and myofibrillar system recovery [11]. During these rearrangements, actin isoforms are shown to have different distribution patterns within the cells and appear to play distinct and non-redundant roles suggesting their spatiotemporal regulation. Despite a lot of data on specific functions of actin isoforms within different muscle and non-muscle cells, the mechanisms regulating their expression and subsequent rearrangements of actin structures are still unclear. At the same time, there is growing evidence to indicate that the dynamics of actin structures in different cell types strongly correlate with the identity and stiffness of extracellular matrix (ECM). In the heart, the composition and distribution of ECM alter during cardiogenesis, being crucial for the cell differentiation process [12,13]. Moreover, ECM proteins were shown to influence the organization of contractile apparatus in CMs in culture [14,15].

The aim of this review is to put together the dynamics of ECM and the contractile apparatus in CMs during the processes of differentiation and adaptation to the culture system and to analyze the role of ECM in the remodeling of actin contractile structures.

## 2. Literature Overview

The maturation of myofibrillar apparatus in CMs during their differentiation has been studied in vivo as well as in vitro [16,17,18,19,20,21,22,23] and is well described (see reviews by Sanger et al. [24,25] for current models of myofibrillogenesis).

Studies on CMs primary culture allowed to reveal the rearrangement of contractile apparatus in mature CMs during their long-term culturing [26,27,28] with the following evidence for re-expression of embryonic isoforms of contractile proteins [2,8,9,29,30,31]. However, the causes and mechanisms of such rearrangements were not well investigated. At the same time the organization of myofibrils in CMs in vitro was shown to depend on the presence of ECM proteins [32,33]. Through the last decade data is accumulating to indicate that the composition, as well as organization of the ECM, influences the dynamics of contractile structures in CMs [14,15,34].

Cardiac ECM has been investigated with different methods. Pinkert et al. overviewed the imaging technics for cardiac ECM [35], and Hacker [36] summarized different animal models with altered expression of genes related to cardiac ECM that reveal the role of individual matrix components. There are some excellent reviews that describe the composition and distribution of ECM in heart tissue [37] and characterize its alterations during heart development [13,38] and aging [39]. The role of ECM turnover in heart physiology as well as heart pathology has been overviewed [40,41,42,43] and the effect of natural as well as synthetic ECM for the proliferation, attachment, and differentiation of different heart cells has been evaluated [44].

There are also some great reviews that look into the cell-matrix communication in the heart, including integrin-mediated mechanotransduction [45,46,47,48]. Studies from modulation of the expression of different integrins in heart tissue have been summarized [49] and the role of integrins in the initial steps of myofibrillogenesis was discussed [50].

However, there is still no available overview to correlate the identity and organization of ECM with the remodeling of actin contractile structures in cardiomyocytes.

## 3. Actin-Based Contractile Systems in Muscle and Non-Muscle Cells

### 3.1. Actin Cytoskeleton

Contractile structures of both muscle and non-muscle cells are based on actin filaments. However, the contractile apparatus of muscle cells, on the one hand, and the actin cytoskeleton of non-muscle cells, on the other hand, are considered to be fundamentally different systems where the structurally stable myofibrillar apparatus of muscle cells is commonly contrasted with the highly dynamic cytoskeletal structures.

Continuous rearrangements of the actin cytoskeleton are provided by rapid polymerization/depolymerization of actin filaments, which can be organized into three main patterns inside the cells, including branched filament networks, filament parallel bundle arrays, or bundle arrays of mixed polarity [51]. These organization types are involved in the formation of more complex structures, composed of polymeric actin and actin-binding proteins [52,53]. The most characteristic actin structures include actin filament network in lamellipodia, actin filament bundles in filopodia, and actin stress fibers [51,52,53], the latter being contractile structures that require cell attachment to extracellular components or other cells. In addition to actin, stress fibers include actin-binding proteins, such as myosin II [54], tropomyosin [55], α-actinin [56], filamin [54], myosin light chain kinase [57], caldesmon [58], and palladin [59]. Some authors believe that the general organization of proteins in stress fibers resembles muscle myofibrils [60]. However, stress fibers lack sarcomeric organization. Filamin is distributed continuously along the actin filament, while myosin II and α-actinin show point periodic distribution and are adjacent to each other. Myosin light chain kinase, caldesmon, and tropomyosin colocalize with myosin, while palladin colocalizes with α-actinin [52,59].

The ability of stress fibers to contract has been shown in living cells, as well as in cells permeabilized with detergent, in the presence of ATP [61,62]. However, unlike muscle myofibrils, characterized by repeated cycles of contraction and relaxation, stress fibers are constantly contracted with irregular acts of relaxation or stretching. In addition, stress fibers contract unevenly over the entire length, and the distance between the myosin or actin containing sites of stress fibers can vary [63].

### 3.2. Actin Filaments in Myofibrillar Apparatus

In contrast to the dynamic cytoskeleton, the highly organized contractile apparatus of muscle cells, in particular CMs, is considered to be a structurally stable system. It is presented by cross striated myofibrils, composed of thick and thin filaments, which are actin and myosin based structures, accordingly.

Thin filaments are formed by fibrillar actin in combination with tropomyosin and troponin complex. Fibrillar actin is a double-stranded helix, each strand of which is formed by globular actin subunits. The tropomyosin molecule consists of two α-helices and is located in grooves of the actin filament. In the absence of calcium, tropomyosin prevents the interaction between myosin and actin fiber. Troponin complexes are located along the thin filament at regular intervals corresponding to the length of the tropomyosin molecule, and are composed of three proteins: troponin I associated with actin, troponin T associated with tropomyosin, and troponin C, which belongs to a class of regulatory proteins called calmodulins and is activated by Ca^2+^ binding. When calcium binds to specific sites of troponin C, tropomyosin releases from the active sites of the actin molecule so as to allow myosin interaction with actin fiber. The thin filament is further stabilized by nebulin protein [64].

Thin and thick filaments are organized into structural and functional units called sarcomeres. The sarcomere length can vary, but usually is about 2 microns. The cross-section of each sarcomere demonstrates two overlapping hexagonal lattices of thick and thin filaments [65]. Thick filaments in sarcomeres have a bipolar organization, while thin filaments are attached by one end to the so-called Z-disk and are of opposite polarity on each side thereof. Z-disks include actin-binding proteins such as α-actinin, filamin, desmin, as well as CapZ proteins that cap fibrillar actin. α-actinin forms transverse bridges between actin filaments, combining them into bundles. Filamin, like α-actinin, interacts with F-actin to form bundles of actin filaments. Both proteins are localized inside Z-disks. In contrast, desmin is located on the periphery of Z-disks, and is also detected in the areas where Z-disks adjoin the plasma membrane. Desmin combines thin filaments of one myofibril and also binds Z-disks of neighboring myofibrils, maintaining their common register [66,67,68].

### 3.3. Actin Isoforms

Muscle and non-muscle contractile systems are based on different actin isoforms. The actin family in vertebrates consists of six closely related proteins [69,70], which are encoded by separate genes and are highly conserved [71,72]. All six functional genes of actin are located on different chromosomes. The genes encoding various actin isoforms have different promoters that are regulated by distinct sets of transcription factors [73]. Depending on the isoelectric point, actins are divided into three classes which are α-, β-, and γ-actins [74,75,76]. β-Actins (β-CYA) and γ-actins (γ-CYA) are characteristic of non-muscle cells [76]. These isoforms are expressed ubiquitously and are known as cytoplasmic actins. In contrast, α-actins are considered to be tissue-specific actin isoforms and are characteristic of muscle cells [77]. Myofibrils of skeletal and cardiac muscles contain different α-isoforms, skeletal α-actin (α-SKA) and cardiac α-actin (α-CAA). Another α-isoform of actin is represented by smooth muscle α-actin (α-SMA), which is characteristic of vascular smooth muscle and myoepithelial cells, and is also found in myofibroblasts. One more tissue-specific actin isoform is smooth muscle γ-actin (γ-SMA), which is mainly expressed in smooth muscles of the intestine and other internal organs.

The maximum amino acid sequence differences of actins isolated from different cells and tissues do not exceed 10% [78], however, cytoplasmic non-muscle actins are more similar to each other than to muscle actins. Four muscle actins differ in 10 out of 375 amino acid residues. α-CAA differs from α-SKA only in four residues in positions 2, 3, 299, and 388. γ-SMA differs from α-SMA in four residues in positions 1, 4, 5, and 360. Cytoplasmic actins differ from muscle actins approximately by 25 amino acid residues and differ from each other only by four residues in positions 1, 2, 3, and 9, all of which are within the 10 N-terminal amino acid residues. There is another classification of actins based on the specificity of N-terminal processing. The most variable region of actin molecules is represented by 10–20 N-terminal amino acid residues. It is believed that this site plays an important role in the regulation of actin polymerization in vivo and in vitro [79]. The N-terminus of the actin molecule is acetylated, and the acetylated amino acid residue (N-acetyl-Asp or N-acetyl-Glu) results from a post-translational multi-step process [80]. Isoactins, called class I molecules, are encoded as polypeptides containing Met-Asp/Glu at the N-terminus. When the N-terminus is removed, the new N-terminal residue (Asp or Glu) is acetylated, providing a mature form of protein. This class combines cytoplasmic β- and γ-actins and smooth muscle γ-actin. Class II molecules are encoded as polypeptides containing Met-Cys-Asp/Glu at the N-terminus. In these actins, acetylation of N-terminal Asp or Glu occurs after a stepwise process, including removal of Met, acetylation of Cys-Asp/Glu, and removal of acetyl-Cys. Class II includes skeletal muscle, cardiac muscle, and smooth muscle α-actin.

Since each actin isoform is encoded by a separate gene, the specificity of their synthesis is regulated at the expression level of corresponding genes. The relationship between cytoskeletal and muscle isoforms is likely to be further regulated by N-terminal processing, the latter being two-step or multi-step, respectively [77].

## 4. Dynamics of Contractile Apparatus in Cardiomyocytes

### 4.1. Myofibrillogenesis

In spite of the fact that muscle vs. non-muscle contractile systems based on different actin isoforms are commonly contrasted to each other, their transition can be observed in heart development, since the precursors of muscle cells are non-muscle cells. Therefore, in the process of CMs differentiation, the contractile apparatus evolves from cytoskeletal structures.

Myofibrillogenesis was studied on embryonic cardiac muscles of different animals, as well as on tissue and cell cultures [18,19,20,21,22,23]. The development of a mammal heart is a multistage process and begins with the specification of CMs precursors and their subsequent differentiation. Precursor cells contain cytoskeletal actin structures distributed close to the cell membrane [81,82]. During myofibrillogenesis, cytoskeleton is replaced by muscle-specific, highly organized myofibrillar apparatus [81].

According to the most common three-step model of myofibrillogenesis, the assembly of myofibrils begins with the association of many sarcomeric proteins into a multicomponent complex [25]. Using electron microscopy, it was shown that the formation of Z-disks begins with the appearance of dense material, which is presented by small membrane-associated complexes, called Z-bodies, connected with actin and myosin filaments. Immunofluorescent studies and electron microscopy have shown the presence of α-actinin and titin in Z-bodies. In this regard, Z-bodies are considered to be precursors of Z-disks [83].

During differentiation of CMs, the sarcomeres increase in size, align and join with each other and with sarcolemma. The first myofibrils, called premyofibrils, are always detected directly under the sarcolemma and then begin to appear in the central part of the cells [18,84,85]. Premiofibrils are composed of sarcomeric proteins, with the exception of non-muscle myosin II, which is gradually replaced by muscle isoform as myofibrils mature [84,86,87,88,89]. Sarcomeres of premyofibrils may have different lengths. As myofibrils mature, the lengths of sarcomeres are aligned, and the neighboring myofibrils are arranged as to allow Z-disks to form a common register. Thus, immature and mature myofibrils can be simultaneously present in the cell. By the end of myofibrillogenesis, densely packed myofibrils fill the most of the cell volume. Mature CMs containing myofibrillar apparatus are called terminally differentiated cells.

### 4.2. Actin Isoform Switching during Differentiation of Cardiomyocytes

Despite the tissue-specific distribution of actin isoforms in adult organisms, during the development of vertebrates, a complex pattern of different isoform expression within the same tissue can be observed [70]. In normal adult myocardium, the contractile apparatus is composed of actin-based myofibrils, where the sarcomeric actin is represented by α-skeletal and α-cardiac isoforms with α-cardiac actin being predominantly expressed [1]. However, in early cardiogenesis, as cells evolve from their non-muscle precursors, the gradual substitution of cytoskeletal beta and gamma actin isoforms for their muscle counterparts occurs [82]. Intensive synthesis of muscle-specific contractile proteins is activated before the formation of myofibrils [90]. During cardiac muscle development, smooth muscle α-actin, which is normally restricted to vascular smooth muscle cells and myofibroblasts, is the first muscle actin isoform [6,7]. As development proceeds, it is sequentially replaced by α-skeletal and α-cardiac actin isoforms [1,2,6,7], which are both expressed in adult myocardium with cardiac α-actin being the main isoform [1,4]. Thus, during differentiation of cardiac muscle cells, coordinated switching of actin isoforms precedes the formation of myofibrils. Different actin isoforms may coexist within the cell as differentiation proceeds.

During stem cell differentiation towards cardiomyocytes [4,5], a replacement of actin isoforms also occurs with transient expression of smooth muscle α-actin [6,7,91]. For example, when CMs are differentiated in vitro from mouse embryonic stem cells, the same pattern of actin isoform expression is observed as that observed in cardiogenesis. In addition, it was shown that inhibition of smooth muscle α-actin expression leads to the impaired differentiation of mouse embryonic stem cells towards CMs [4].

### 4.3. Rearrangements of Contractile Apparatus in Cardiomyocytes in Culture

In spite of the fact that mature CMs are called terminally differentiated cells, their contractile apparatus may rearrange during cell adaptation to the culture system. It is well known that neonatal, as well as adult, CMs are readily transferred into monolayer cultures. However, their long-term culturing is accompanied by significant changes in cell morphology and organization, where the reversible rearrangement of their contractile apparatus occurs with the conversion of typical myofibrils into non-striated structures of non-muscle type and the loss of contractility [2,8,9,14,28,29,30,31]. Non-striated structures are referred to as stress fiber-like structures, since they resemble stress-fibers of non-muscle cells [27,92].

Interestingly, the phenomenon of rearrangement is accompanied by transient replacement of the inherent α-cardiac actin with α-smooth-muscle isoform [2,8,9,10,11]. Since smooth muscle α-actin is characteristic of embryonic CMs, many researchers consider it to be a marker of dedifferentiation of these cells [93,94]. In this regard, the changes observed during the culturing of CMs are often considered as the process of dedifferentiation or the return of cells to the embryonic phenotype. Our previous results show that the appearance of smooth muscle α-actin precedes the transformation of myofibrils into structures of non-muscle type and corresponds to the inhibition of contractile activity. Interestingly, myofibrils that are still present in the cells are intensely stained with antibodies against smooth muscle actin along with non-striated structures [11]. To date, quite a lot of data has accumulated to indicate that different actin isoforms, despite conservative sequences, cannot replace each other without affecting thefunction [95,96,97,98,99,100,101]. In particular, smooth muscle and skeletal actin isoforms cannot ensure the normal formation of myofibrillar apparatus in cardiac isoform knockout models [98]. In light of these data, the inclusion of smooth muscle α-actin into myofibrillar structures when CMs are transferred into culture system seems to result in the inhibition of CMs contractile activity and transformation of myofibrils into structures resembling stress fibers of non-muscle cells. The fact that smooth muscle α-actin is the main protein of stress fibers in myofibroblasts [102,103] supports that stress fiber-like organization of the contractile system may be more preferable for this actin isoform. The incompatibility of smooth muscle actin with myofibrillar organization is also confirmed by the release of sarcomeric proteins from actin-containing structures into cell cytoplasm during the rearrangement of myofibrillar apparatus in cultured CMs [104]. It was shown that actin-binding proteins may distinctively interact with certain actin isoforms and contribute to the functional specificity of different actins [3,77,105,106,107,108,109,110,111,112,113].

The rearrangement of the contractile apparatus is followed by the restoration of myofibrillar system and the recovery of contractility. These changes are accompanied by a decrease in smooth muscle actin isoform, which leaves the area occupied by the newly formed myofibrils. Interestingly, at the initial stages of myofibrillar apparatus recovery, a small amount of smooth muscle actin is detected in some fragments of nascent myofibrils, suggesting that myofibrils are formed by gradual replacement of smooth muscle isoform with cardiac actin [11].

Thus, the rearrangements observed in CM primary culture may be considered as a dedifferentiation process followed by the maturation of myofibrillar apparatus. During rearrangements of the contractile system in CMs in vivo and in vitro, actin isoforms are shown to have different distribution patterns within the cells and appear to play distinct roles, suggesting their spatiotemporal regulation.

Despite a lot of data on the specific functions of actin isoforms within different muscle and non-muscle cells, the causes and mechanisms regulating their expression and subsequent remodeling of actin structures are still unclear.

For this, the rearrangement of the contractile apparatus in CMs in culture may be a clue as to how actin dynamics are regulated. When transferred to the culture system, cells lose their microenvironment and the ability to maintain the initial organization of the contractile system. However, the reversible nature of rearrangement indicates that during long-term culturing CMs are able to reconstitute the microenvironmental cues required for the maturation of their myofibrillar apparatus.

## 5. Extracellular Matrix

### 5.1. Cardiac Extracellular Matrix

Substantially all cells in an organism are surrounded by extracellular matrix (ECM) which is an organized spatial network of macromolecules secreted by cells that provide structural and biochemical support [114,115,116]. It has been shown that cardiac ECM is necessary for cell migration, proliferation, and differentiation [117] and is important for the structural integrity and elasticity of heart tissue providing mechanical stiffness [39,118]. Myocardial ECM is composed of collagens, glycoproteins (e.g., fibronectins, elastin, laminins) and proteoglycans [119]. Collagens are the most abundant structural component of ECM in the heart [120]. Collagens can be divided into two major classes, the fibrillar and non-fibrillar collagens [121]. Five collagens (collagens I, II, III, V, and XI) form fibrils [121]. The fibril-forming collagens provide the tissue structural framework [119,121,122]. In the heart, type I collagen is the main component of ECM. It makes up about 85% of all collagens in the heart, while collagen III is about 11% [123]. In vivo, collagen I assembles into aligned fibers and regulates heart growth [124,125].

Another important component of myocardial ECM is fibronectin, which has been implicated in cell adhesion, being crucial for cell binding to other ECM components. Fibronectin was shown to be essential for heart development and repair [44].

Elastin is one more important component of cardiac ECM, which regulates the elasticity of cardiac tissue [126]. Elastin has been shown to be essential for the proper development of the heart and vasculature [127].

In addition to the general ECM, cardiomyocytes have their own basement membrane which is a highly organized layer of proteins on the outer surface of sarcolemma composed of glycoproteins and proteoglycans, such as collagen IV, laminins, entactins, perlecan, and chondroitinsulfate [128,129,130,131]. Components of the basement membrane are known to play a role in both cardiac tissue stabilization and angiogenesis and are the first extracellular proteins synthesized during embryogenesis [132].

For a detailed description of ECM composition and organization in the heart see excellent reviews [13,39,48].

In heart tissue, fibroblasts and smooth muscle cells are the main producers of ECM components, including fibronectin, laminin and collagen type I, III, and IV. However, the ability to synthesize components of the basement membrane was shown for endothelial cells, as well as for cardiac myocytes in vitro [37,129,133]. Recent studies indicate that ECM components can also be synthesized by the stem cells of the heart [134].

### 5.2. Extracellular Matrix in Heart Development

Cardiac ECM plays an important role in embryogenesis [135]. The distinct roles of different ECM proteins in heart development have been approved in vivo using knockout experimental animal models (summarized in Table 1). For example, in mice, mutations in collagen I were shown to impair heart development [136], and collagen III knockout resulted in life-shortening due to vessel rupture [137]. Lack of the fibronectin gene was lethal in early embryogenesis resulting in various cardiac and vascular defects [138]. Lack of the elastin gene resulted in mortality several days after birth [139]. Collagen IV knockout did not impair heart development until embryonic day 9.5, but was lethal in following days due to structural defects in basement membrane [140]. Deletions of the laminin α1 chain were shown to be lethal at early embryonic stages [141], whereas deletions of the laminin α4 chain led to impaired microvessel maturation [142].

The composition and distribution of ECM proteins were shown to alter during cardiogenesis, being crucial for the cell differentiation process [12,13]. Changes in collagen and other ECM proteins are most evident during the rapid growth of a neonatal heart [157]. In general, the level of all ECM proteins is strongly decreased in the adult heart [158,159].

Type 1 collagen predominates at each stage of heart development [160]. In mouse hearts, the amount of collagen I was greatest at an early embryonic stage, however, an increase in the density and organizational complexity of collagen fibers was observed from embryonic to postnatal development [161]. During early development of chicken heart, expression of collagens I and III remained stable throughout the late stages of fetal growth [162]. In hamster heart, collagen synthesis was elevated during neonatal development especially the first 4–5 days after birth [163]. In rat hearts, ventricular expression of types I and III collagen genes was shown to reach its maximum within the first 2–3 postnatal weeks with the following rapid decline [164].

The ratio of collagen I to collagen III is high in relatively stiff neonatal hearts, however, for some time after birth it decreases and becomes stable in adulthood [126]. Besides the ratio of collagens their crosslinking was shown to contribute to the heart stiffness (see, for example, a recent review by González et al. [165]). In general, cardiac tissue is becoming less compliant during heart development [166].

The expression of total fibronectin in rat myocardium is high during embryogenesis and decreases in postnatal life. In adult rat hearts, it is ten-fold less as compared to the fetus [159]. In humans, fibronectin expression is relatively constant during fetal life, but decreases after birth [167].

Elastin levels in mouse heart are low early in development but increase significantly from embryonic to postnatal life [161,164]. The dynamics of elastin synthesis seems to be essential for the regulation of elasticity of cardiac tissue [126]. Development of the cardiac basement membrane plays a key role in the organogenesis of the myocardium, where basement membrane components are the first extracellular proteins synthesized during embryogenesis [132]. Collagen IV in mouse hearts is found throughout the development and increases in amount from early embryonic to early postnatal stages [164]. Laminin expression in humans was shown to be not age-dependent and constant throughout life [167]. However, the remarkable changes in spatial distribution of laminin during development of rat hearts were shown [168].

### 5.3. Extracellular Matrix in Cardiogenic Differentiation Methods

Because of the established role of ECM in cardiogenesis, some methods of cardiogenic differentiation include ECM proteins as a scaffold to enhance differentiation of various cells towards CMs. The enhanced expression of cardio-specific markers was demonstrated in cardiomyocyte-like cells derived from mesenchymal stem cells cultured on collagen V as compared to collagen I and the lack of matrix [169]. In CMs, spontaneously differentiated from mouse embryonic stem cells the cardiac fibroblast-derived ECM supported earlier cell maturation as compared to the commercial ECM (Matrigel) and the lack of ECM [170]. Culturing of mouse embryonic stem cells on thin sections of decellularized heart tissue induced high expression of cardiac myosin heavy chain and cardiac troponin I as compared to cells cultured on liver ECM [171]. Moreover, culturing of human adipose tissue-derived stem cells on the plates coated with the ECM, produced from decellularized heart tissue, induced high expression of cardio-specific genes [172].

Monocomponent, as well as multicomponent, ECM systems, have also been used in 3D in cardiogenic differentiation methods to approximate the natural conditions. The maturation of CMs derived from induced pluripotent stem cells was enhanced when the cells were seeded into a 3D cardiac ECM scaffold as compared to 2D culture [173]. In one study, fibrin gels supported cardiac differentiation in cardiac reprogramming method, whereas Matrigel and collagen I gels were poorly efficient [174]. 3D cardiac fibroblast-derived ECM [175] was shown to control differentiation of bone marrow-derived stem cells toward a cardiomyocyte phenotype [176]. Native ECM, obtained by heart decellularization, was also described to maintain the differentiated state of cardiomyocytes derived from human induced pluripotent stem cells as well as their capability of forming functionally active myocardial segments [177].

Besides the identity of ECM its elasticity, in particular, the concentration of collagen, was shown to influence cardiogenic differentiation. For example, too high a content of collagen reduced the differentiation of human embryonic stem cells towards CMs [178]. In another work a cell-derived ECM cross-linked with the naturally derived cross-linker to provide an elastic modulus approximating the stiffness of the neonatal rat heart was supportive of cardiomyocyte differentiation compared to the uncross-linked ECM [179]. The native ECM, combined with fibrin to adjust matrix stiffness to the mechanical properties of the native myocardium promoted differentiation of cardiac progenitor cells toward CMs [180].

In the light of described data, ECM is emerging as an important regulator of cardiogenic differentiation in vivo and in vitro. On the other hand, there is growing evidence on cell cultures to indicate that identity and stiffness of ECM strongly correlate with the dynamics of actin structures in different cell types.

### 5.4. Culturing of Cardiomyocytes in the Presence of Extracellular Matrix

It is well-known that ECM proteins have a significant effect on the formation of actin cytoskeletal structures in non-muscle cells [181]. The results demonstrate the specificity of the actin cytoskeleton organization in the same cells cultured on different ECM proteins. For example, studies on A431 cells demonstrated differences in cytoskeleton organization for cells cultured on fibronectin and laminin [182]. Similar results were shown for fibroblasts that were cultured on fibronectin, laminin, and type III collagen [183]. Moreover, ECM topography was shown to influence the dynamics of actin cytoskeleton [184].

Much fewer data are currently available on the culturing of CMs in the presence of ECM components. However, there is evidence that matrix identity influences the behavior of CMs in vitro. For example, embryonic CMs, which were cultured on laminin, tended to remain a rod-shaped morphology, whereas CMs, cultured on collagen, became round and flattened in a few hours [185]. In another study, rat neonatal cardiomyocytes were shown to differ in their rate of maturation in culture depending on the type of ECM substrate, where the fibroblast-derived ECM supported earliest maturation in terms of spontaneous contractions, calcium handling efficiency, cell size, and development of the mitochondrion, as compared to commercial laminin and fibronectin [186].

Interestingly, cardiac myocytes isolated from different stages of heart development demonstrated different affinities for specific ECM components. Neonatal as well as fetal myocytes were shown to attach with high affinity to all types of collagen (type I, II, III, IV, and V) and to fibronectin and laminin, with the rate of attachment correlating with the protein concentration [187,188,189]. Myocytes from adult hearts attached efficiently to basement membrane components laminin and type IV collagen in a concentration-dependent manner, however, they demonstrated low attachment to fibronectin and did not attach to interstitial collagens. [187,188,190]. These data led to the hypothesis that recognition of components of the ECM is developmentally regulated [188,191].

Besides matrix composition, its elasticity was shown to influence the contractility of cardiomyocytes. In one study, embryonic cardiomyocytes were shown to beat best on a matrix with heart-like elasticity [192]. In another work, neonatal CMs were cultured on polyacrylamide coated with the type I collagen in various concentrations. The substrate with the elasticity characteristic of the heart tissue was optimal [193]. As stiffness increased, cells ceased to contract, while culturing the cells in a softer substrate reduced the contraction force [193].

There are also some works demonstrating the influence of ECM on the organization of the contractile system in CMs. In one paper, CMs placed on different ECM proteins exhibited different patterns of myofibril distribution. Cells on collagen I and III contained striated myofibrils which extended to the cell perimeters where focal adhesions were predominately located. In the cells plated on laminin and fibronectin myofibrils and focal adhesions were located more centrally. In addition, cells on laminin contained circumferential arcs of filaments near the cell periphery [32]. In another study, when single cardiomyocytes were cultured on micropatterned islands, unique myofibrillar patterns were formed with respect to geometric cues in the ECM [33]. The results of our previous work demonstrate that culturing of rat neonatal CMs on individual ECM proteins, such as fibronectin and laminin, remarkably shorten the time of rearrangement of their contractile apparatus [14]. In another study, neonatal cardiomyocytes that were cultured on a naturally occurring ECM, synthesized by cardiac fibroblasts, exhibited spontaneous contractility earlier with the earlier maturation of their myofibrillar apparatus as compared to cardiomyocytes grown on laminin or fibronectin alone [175]. These results may reflect the synergistic effect of numerous components in fibroblast-derived ECM [175]. When rat neonatal CMs were cultured in 3D collagen gels, the rearrangement of their myofibrillar apparatus did not occur, however, their contractility was impaired [15]. Different concentrations of collagen I in 3D gels resulted in differences in cell morphology and myofibril organization [15]. The effect of matrix stiffness on the contractile apparatus was also shown for rat neonatal CMs cultured on collagen-coated polyacrylamide gels. Cells on the substrate with myocardium-like stiffness formed aligned sarcomeres in contrast to CMs cultured on the stiffer substrates which exhibited unaligned sarcomeres and stress fiber-like structures [194].

In general, data on the culturing of CMs in the presence of ECM components indicate that ECM is an important regulator of not only the cytoskeletal structures in non-muscle cells, but also of the contractile apparatus in muscle cells.

### 5.5. Extracellular Matrix Production by Cardiomyocytes in Culture

The effect of ECM on the occurrence and duration of the contractile apparatus rearrangement in cultured CMs suggests that it is the lack of ECM that leads to the CMs dedifferentiation when they are transferred to the culture system. In the heart tissue, non-muscle cells, such as fibroblasts and endothelial cells, are responsible for the synthesis of ECM, while CMs specialize in the contraction. However, it was shown that CMs synthesize their own basement membrane components when placed into culture system. For example, rat neonatal CMs cultured on aligned collagen substrates for 3 days in vitro produced a basement membrane component laminin, where its deposition changed from 4 to 72 h in culture from a dot-like shape to a fine fishnet-like network [32]. Another study has shown that adult rat CMs cultured in vitro synthesize basement membrane components collagen type VI and laminin as they progress into culture over a 14 day period. The deposition patterns of the collagen type IV and laminin were different with the laminin forming a dense layer beneath the cells and the collagen type IV forming a much finer network [129]. Previously we have shown that, besides basement membrane components, rat neonatal CMs in culture begin to synthesize structural components of cardiac ECM collagen type I [11]. Interestingly, a strong correlation between the ECM production and the dynamics of contractile system was shown during long-term culturing of CMs. The gradual accumulation of ECM proteins was observed in CMs culture with their maximum level corresponding to the stage of contractile apparatus rearrangement and the loss of contractility. This maximum accumulation preceded the recovery of myofibrillar organization, suggesting that it is the ECM acquired by this time that is necessary and sufficient for the restoration of myofibrillar system. These data allowed to speak of a temporary change in CMs function in the process of adaptation to monolayer culture system from contractile to non-typical secretory. In this regard, the reorganization of contractile apparatus with the transformation of myofibrils into structures of non-muscle type seems to be a necessary condition for CMs to synthesize their own ECM components, since a highly organized myofibrillar apparatus, which occupies the most of cell volume, can interfere with the active synthetic processes. The decrease in ECM production following the restoration of CMs contractile apparatus and the recovery of contractility confirms the return of cells to their initial function [11].

These results are particularly interesting when they are considered together with the data on actin isoform switching in CMs. The ECM accumulation in CMs culture goes along with α-smooth muscle actin downregulation and precedes the upregulation of α-cardiac actin expression [11]. These data may suggest a feedback loop between ECM and actin isoforms expression [11], allowing to speak about the regulation of actin system dynamics in CMs by ECM.

## 6. Integrins in Cardiomyocytes

It is well known that the interaction of cells with ECM components is mediated by integrins [195,196,197], which are transmembrane receptors connected with the ECM components by their extracellular domains and with the cell contractile structures via intracellular domains. Integrins are expressed in all cell types and consist of alpha-beta heterodimer units. In mammals, more than 18 α and 8 β subunits were identified, which can combine to form at least 24 distinct receptors. It has been shown that integrins can transmit signals from the extracellular space into cells via mechanotransduction, which is the process of converting mechanical forces (in particular, ECM tension) into biochemical cues [49,198,199]. Because integrins do not possess enzymatic activity, they must trigger downstream molecules to transmit their signals within the cell [195,200,201]. Activation of integrins is followed by their clustering with the attraction of adapter proteins and non-receptor kinases to their cytoplasmic domains [202]. Using this mechanism, extracellular signals can be translated into appropriate cellular responses, such as migration, differentiation, growth, and survival, as well as tissue remodeling [49,198,199]. The role of integrins as mechanoreceptors has been described for various cell types, including cells of cardiovascular system [203,204,205]. It was shown that integrins are necessary for the normal development and functioning of the heart and can participate in the regulation of protein expression and synthesis in heart cells [45,200,206,207].

In CMs, α1β1, α5β1, and α7β1 are the most abundant integrin heterodimers that bind mainly collagen, fibronectin, and laminin, respectively. The main β integrin subunit is β1, which is predominantly expressed in costameres of cardiac myocytes, where the cells attach to the collagen network [208]. It was shown that cardiomyocyte differentiation, particularly organization of sarcomere structures, is crucially dependent on the presence of βl integrin [209].

In addition to variations in subunits, the integrin repertoire is extended by alternative splicing. For example, β1 integrin has four isoforms. In the heart, the isoform β1A is expressed by non-muscle cells, and the β1D is expressed in mature cardiomyocytes [208,210], with these isoforms differing in their exchange dynamics and adaptor proteins recruitment [211]. α7 integrin subunit also has multiple alternatively-spliced variants with the α7B being the major partner for β1D isoform in cardiomyocytes of adult heart [208,210].

Some studies imply that cardiomyocytes on different stages of development have different integrin receptors suggesting that integrin expression is developmentally regulated [212]. During cardiogenesis, splice variant β1D is known to replace the common isoform β1A, which is predominantly expressed in the embryo [208,213]. Similar to β subunits, expression of α-chains varies with the stage of development [214,215,216]. In fetal as well as neonatal CMs, α5 subunit is mainly expressed. However, with the onset of postnatal development it is replaced by α7, which is the main α subunit in adult CMs [214]. Moreover, in fetal as well as neonatal myocytes isolated from rat hearts the expression of α1 and α3 subunits was demonstrated, whereas freshly isolated cells of adult hearts were shown to lack α1 chain. [189]. In general, the expression of α1 chain was observed at stages of increased collagen synthesis [189].

Interestingly, changes in integrin distribution were shown in the adaptation of rat neonatal CMs to culture system. The cellular localization of α3βl integrin dramatically changed from a diffuse distribution to a sarcomeric banding pattern during the maturation of contractile apparatus, and its localization in sarcolemmal regions associated with Z-disks closely correlated with myofibril assembly and organization of sarcomere structures [189,191]. Moreover, the addition of antibodies against β1 integrin to CMs cultured on different ECM components impaired cell spreading and myofibrillogenesis [217]. These data suggest that integrin dynamics are likely to be linked with the development of contractile apparatus in CMs.

The mechanisms by which the engagement of different integrins results in remodeling of contractile structures are not described. However, relevant knowledge has accumulated through the last decade that allows to contemplate them.

## 7. Possible Mechanisms of Integrin-Mediated Regulation of Actin Contractile System in Cardiomyocytes

**Figure 1:**
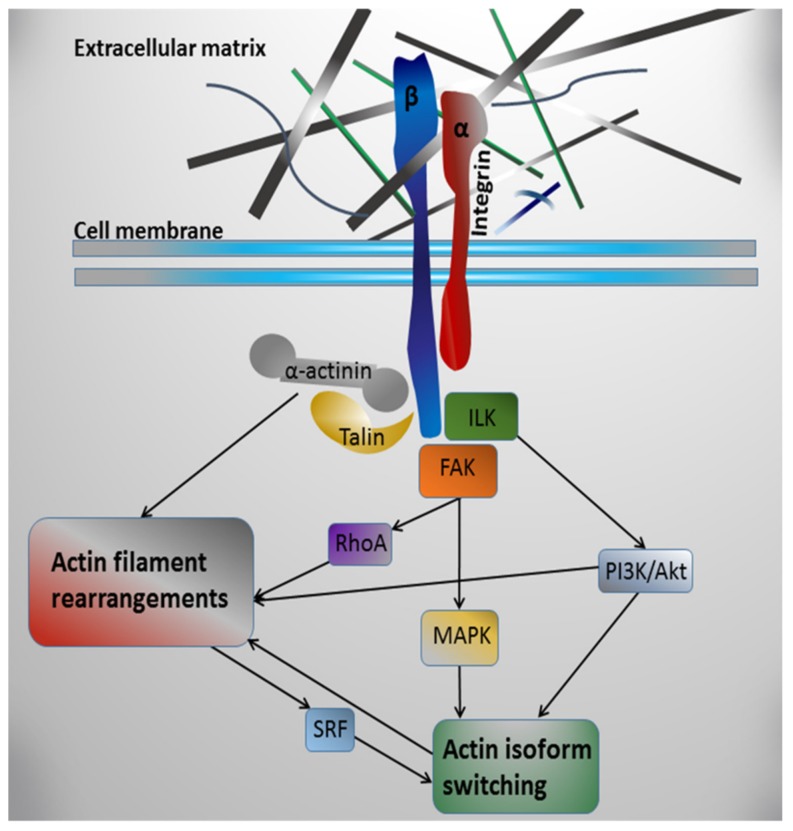
Schematic diagram summarizing the possible mechanisms for integrin-mediated regulation of actin filament rearrangements by extracellular matrix in cardiomyocytes.

Integrin receptors are linked with actin contractile structures via direct interaction of their cytoplasmic domains with actin-binding proteins, such as talin [218] and α-actinin [219]. Besides this mechanical linkage, integrins can affect microfilament system dynamics via the attraction of non-receptor kinases to their cytoplasmic domains after interaction with ECM components [202] followed by the activation of relevant signaling pathways [220,221,222] (see Figure 1). One of the tyrosine kinases, activated by integrin binding with ECM proteins, is the integrin-linked kinase (ILK) [223,224,225]. ILK was shown to mediate actin filament rearrangements through PI3K/Akt/Rac1 signaling [226]. Moreover, ILK activation has been linked to α-SMA expression in some models [227]. Inhibition of ILK expression in normal dermal fibroblasts suppressed α-SMA expression likely through ILK-PI3K/Akt signaling pathway [228]. In rat neonatal CMs, the rearrangements of their contractile apparatus in culture were accompanied by changes in ILK level and its redistribution from perinuclear area to a sarcomeric pattern [229].

Another kinase attracted by integrins is the focal adhesion kinase (FAK). It is believed that FAK plays a key role in the further transfer of intracellular signals after the activation of integrins [230,231,232]. In vitro studies have indicated that, in rat neonatal cardiomyocytes, the interaction of cytoplasmic integrin domains with FAK can mediate the phosphorylation of mitogen-activated protein kinases (MAP kinases), such as ERK, p38, and JNK, [233], where p38 was shown to regulate α-SMA promoter activity in smooth muscle cells and to be a crucial mediator of mechanical force-induced α-SMA expression in fibroblasts [234,235]. Besides MAP kinases, the interaction of cytoplasmic integrin domains with FAK [236,237] may lead to the recruitment of Rho family GTPases, which subsequently regulate key steps in actin cytoskeleton polymerization and reorganization [238]. The small GTPase RhoA is involved in many actin-based cytoskeletal processes, including formation of stress fibers, cell adhesion, cytokinesis, and contractility [239,240]. RhoA activation was shown to cause stress fiber formation and integrin clustering with associated proteins into focal adhesion complexes [241].

Interestingly, actin rearrangements themselves might be involved in the regulation of actin gene expression (see Figure 1). It has been shown that the dynamics of cytoplasmic actin can affect the activity of transcription factors and, thus, modulate the expression of various genes [242,243]. The best known actin-regulated transcription factor is serum response factor (SRF), which controls the expression of many genes associated with contractile structures in response to the relative concentration of actin filaments and actin monomers [244]. SRF was shown to be necessary for the expression of skeletal, cardiac, and smooth muscle α-actin genes [49,241,245,246,247] and is known to be an important regulator of force-induced smooth muscle actin (SMA) expression [248].

Taken together, described data allow to suggest that integrin-mediated mechanotransduction could control the dynamics of actin contractile apparatus in CMs via the activation of signaling molecules involved in regulation of actin isoforms and rearrangement of actin microfilament system.

## 8. Conclusions

The obvious correlation between the organization of actin structures and actin isoforms expression in CMs, on the one hand, and ECM expression and organization, on the other hand, suggests the regulation of actin dynamics by ECM. The mechanisms underlying are likely to be mediated by transmembrane integrin receptors (see Figure 1). However, the in vivo investigation of integrin engagement and following signaling pathways is limited by the complex organization of heart tissue, which makes it difficult to isolate the effects of individual ECM proteins on particular cell processes [44]. In vitro investigations on the cells induced for cardiogenic differentiation are still ineffective because of the heterogeneity of cell population varying in lineages and stages of differentiation. Moreover, cardiogenic inducers can mask the intrinsic effects of matrix components. The primary culture of CMs appears, therefore, to be a better solution being a homogenous culture of cardiac muscle cells reproducing the processes of contractile apparatus dedifferentiation and redifferentiation in vitro. The advantage of this system is the ability to better distill the interplay of cells with particular ECM components and to discern the signaling pathways elicited during rearrangements of contractile structures. Such investigations may shed a light on the ECM-dependent regulation of contractile system in CMs during the key physiological as well as pathological processes.

## Figures and Tables

**Table 1:** Mouse models with altered gene expression demonstrating the role of individual extracellular matrix proteins in heart development.

Extracellular Matrix Protein	Model	Effect on Cardiovascular System	Reference
Collagen I	Col1a2-deficient mice	impaired heart development, decreased heart weight, altered mechanical and structural properties of the ventricular myocardium	[136]
	Col1a1-deficient mice	vascular abnormalities, age-dependent aortic dissection and rupture	[143,144]
	Col1a1^−/−^ mice	normal development up to embryonic day 12, lethality between embryonic days 12-14 due to rupture of major blood vessels	[145]
Collagen III	Col3a1^−/−^ mice	abnormal cardiac development, life-shortening due to rupture of major blood vessels	[137]
Collagen IV	Col4a1/2^−/−^ mice	structural defects in the basement membrane, lethality between embryonic days 10.5-11.5 due to pericardial bleeding and rupture of major blood vessels	[140]
Collagen V	Col5a1^−/−^ mice	lethality at embryonic day 10 due to cardiovascular insufficiency	[146]
Collagen XI	Col11a1^–/–^ mice	lethality at birth, thickening of the interventricular septum and atrioventricular valve leaflets, significant changes in the heart shape	[147]
Col XV	Col15a1^−/−^ mice	defects in vessel architecture, impaired microvascular hemodynamics, defects in heart structure and function	[148,149]
Collagen XVIII	Col18a1^−/−^ mice	significant thickening of the endothelial basement membrane in the atrioventricular valves of the heart	[150]
Fibronectin	Fn1^−/−^ mice	multiple developmental abnormalities at embryonic day 8, lethality at embryonic day 10 due to cardiac and vascular defects	[138,151]
	EIIIA^−/−^ or EIIIB^−/−^ mice	normal phenotype, viability, and fertility without defects in angiogenesis	[152]
	EIIIA^−/−^ EIIIB^−/−^ mice	severe cardiovascular defects by embryonic day 9.5, including vascular hemorrhage, impaired angiogenesis and heart defects, lethal at embryonic day 10.5	[153]
Elastin	Eln^−/−^ mice	lethality at day 4.5 of postnatal development due to obstructive arterial disease	[139]
	Eln^+/^^−^ mice	changes in the arterial wall structure, high blood pressure	[154]
Laminin	Lama1^−/−^ mice	lethality after embryonic day 6.5 due to defects in the extraembryonic basement membrane	[155]
	Lama4^−/−^ mice	hemorrhages during the embryonic and neonatal development, impaired microvessel maturation, ischemic cardiac phenotype	[142,156]

## Abbreviations

CMs	Cardiomyocytes
ECM	Extracellular matrix
SMA	Smooth muscle actin
ILK	Integrin-linked kinase
FAK	Focal adhesion kinase
SRF	Serum response factor
